# The Functional Evaluation of Eating Difficulties Scale: Study Protocol and Validation in Infants with Neurodevelopmental Impairments and Disabilities

**DOI:** 10.3389/fped.2017.00273

**Published:** 2017-12-18

**Authors:** Anna Cavallini, Livio Provenzi, Daniela Sacchi, Laura Longoni, Renato Borgatti

**Affiliations:** ^1^Neuropsychiatry and Neurorehabilitation Unit, Scientific Institute IRCCS Eugenio Medea, Bosisio Parini, Italy; ^2^0-3 Center for the At-Risk Infant, Scientific Institute IRCCS Eugenio Medea, Bosisio Parini, Italy

**Keywords:** developmental disabilities, feeding disorders, infants, oral-motor skills, protocol, rehabilitation medicine

## Abstract

**Introduction:**

A reliable and accurate evaluation of oral-motor skills in newborns at risk for swallowing and feeding disorders is key to set the goals of effective early interventions. Although many tools are available to assess oral-motor skills in newborns, limited evidence exists for what pertains their reliability and their effectivity in predicting short- and long-term developmental outcomes in at-risk infants. The aim of the present study is to develop and provide a preliminary validation of a new clinically grounded tool [i.e., the Functional Evaluation of Eating Difficulties Scale (FEEDS)] specifically designed to be used with at-risk newborns and infants. The paper describes the steps of tool development and information on the reliability of the tool are provided.

**Methods/analysis:**

The FEEDS has been developed according to clinical evidence and expertise by a multidisciplinary team of professionals dealing with feeding problems in at-risk infants diagnosed with neurodevelopmental impairments and disabilities. The steps of FEEDS development are reported, together with a detailed description of items, scoring procedure, and clinical cutoff. The FEEDS has been applied to a relatively large sample of 0- to 12-month-old infants (*N* = 136) with neurodevelopmental disability, enrolled consecutively between 2004 and 2016 at the Scientific Institute IRCCS Eugenio Medea (Bosisio Parini, Italy), which is the main rehabilitation hospital for children with neurodevelopmental disabilities in Italy. Internal consistency (Cronbach’s alpha) and reliability (inter-rater agreement) have been assessed.

**Ethics and dissemination:**

All the procedures are consistent with the World Medical Association Declaration of Helsinki (2013) and the FEEDS has been approved by the clinical committee of the Scientific Institute IRCCS Eugenio Medea. Further psychometric characteristics and evidence of the predictive validity of the FEEDS will be obtained on a larger sample and they will be reported in future publications from this group.

## Introduction

The development of autonomous swallowing and feeding involves both reflexive and voluntary motor control and sensory processing and constitutes a key milestone of early oral-motor development (1). The act of feeding and swallowing includes three phases: (1) an oral phase during which the bolus is prepared and transported to the pharynx; (2) a pharyngeal phase during which the swallow is triggered and the bolus moves throughout the pharynx; and (3) the esophageal phase during which the bolus arrives to the stomach and digestion starts.

Feeding and swallowing disorders include delays and/or difficulties in the development of autonomous swallowing and feeding (i.e., eating and drinking skills) and they are common in pediatric populations diagnosed with early neurodevelopmental disorders and psychomotor delay (e.g., prematurity, neurological diseases, genetic disorders, severe perinatal injuries). Notably, the transition to autonomous feeding is considered among golden standard criteria for hospital discharge in at-risk infants and children, together with neurobehavioral stability and adequate weight gain rate (2). Moreover, the presence of feeding and swallowing disorders have a relevant impact on the developmental trajectories of the child (3), on parental well-being (4, 5) and on the quality of early parent–child interaction, which is considered as the main proxy for optimal developmental outcomes (6).

In order to adequately treat feeding and swallowing disorders, an accurate and precocious evaluation of functional oral-motor skills is needed and is key to rehabilitation success, as it allows clinicians to identify, monitor, and manage feeding problems through individualized rehabilitation programs (7). Many diverse assessment tools for early feeding and swallowing disorders have been developed over the years (8). Nonetheless, there is no general consensus on the better selection for feeding assessment in at-risk and clinical pediatric populations. Howe and colleagues (8) provided a comprehensive overview of available tools highlighting different dimensions of scientific integrity (e.g., reliability and validity). Complete information about the psychometric properties of the included tools were not always available and both reliability and validity were rated highly in a limited subset of assessment instruments and the review findings were inconclusive. The Neonatal Oral-Motor Assessment Scale (NOMAS) emerged as the most thoroughly used and tested instrument. Nonetheless, specific limitations of the NOMAS emerged (9, 10). Limited information on the reliability of the NOMAS was available; on the one hand, moderate reliability emerged among three studies, on the other hand, different scoring methods (e.g., occurrence rate, qualitative scores, numbers of at-risk behaviors) were used within the retrieved literature. Similarly, mixed findings and moderate capacity to predict further feeding developmental outcomes in older infants and children have been reported for the NOMAS (8)^1^. For example, infants classified as poor feeders might have no difficulties on the oral-motor domain at long-term follow-up evaluations (10). In addition, the specific focus of the NOMAS on the biomechanical components of successful feeding make it limitedly useful to obtain broader information on different aspects of feeding, including maternal–infant interaction processes and infants’ behavioral states during feeding (8).

### Aims of the Present Study

Consistently, the aim of the present study is to present the study protocol of the development of an innovative scientific sound and clinically grounded tool to assess oral-motor skills, feeding, and swallowing disorders in at-risk newborns and infants, namely the Functional Evaluation of Eating Difficulties Scale (FEEDS). This tool has been specifically developed to be applied to infants with neurodevelopmental disorders which need a specific evaluation of the safety and have a necessity of oral-motor rehabilitation and feeding initiation. All the newborns and infants who present complex neurodevelopmental conditions characterized by biomechanical impairment of swallowing and feeding are eligible to be assessed with this tool. Here, we report on (1) item production and selection; (2) administration procedural guidelines; (3) coding system; (4) scientific integrity, including an assessment of reliability, validity, and predictive capacity of the FEEDS in clinical populations of newborns and infants with neurodevelopmental disabilities.

## Methods and Analysis

### Notes on the Setting of the FEEDS Protocol

The FEEDS is a protocoled assessment tool to evaluate early newborns and infants’ abilities to swallow and feed. This tool has been developed at the Department of Child and Adolescent Neurology and Psychiatry of the Scientific Institute IRCCS Eugenio Medea, by a multidisciplinary team made up of a developmental neuropsychiatrist, a phoniatrist and a speech therapist who have extensive and long-lasting expertise in diagnosing and treating early feeding and swallowing disorders in newborns and infants affected by neurodevelopmental disabilities (e.g., severe prematurity, cerebral palsy, genetic and metabolic syndromes, pediatric tumors, etc.). The Scientific Institute IRCCS Eugenio Medea is the main nationally acknowledged child rehabilitation and research institute in Italy and receives patients from the entire Italian country.

In the following paragraphs, we report the development of the FEEDS checklist (Phase 1) and we provide an assessment of FEEDS internal consistency, factorial structure, and reliability (Phase 2).

### Phase 1: The FEEDS Checklist Development

#### Preliminary Assessment of Infants’ Clinical and Behavioral State

Before the FEEDS is administered, specific variables are meant to be noted and registered by the clinician through the observation of the infant and/or based on parental reports. These variables include feeding mode (e.g., nasogastric tube, percutaneous endoscopic gastrostomy (PEG) or percutaneous endoscopic trans-gastric jejunostomy (PEG-J), mixed or others), state of respiration (e.g., autonomous, partial or total mechanical support, tracheotomy), need of secretions’ aspiration (e.g., non-necessary, seldom, frequent) and the behavioral state (e.g., available to interact, unavailable to interact, asleep) (see Table 1).

**Table 1:** Set of preliminary observations.

Domains	Option 1	Option 2	Option 3
Nutrition	□ NG-tubes	□ PEG □ PEGJ □ Nissen	□ mixed

Respiration	□ Autonomous □ Partial oxygen therapy □ Total oxygen therapy	□ Tracheotomy	□ Gurgling

Secretions’ aspiration	□ Unnecessary	□ Seldom	□ Frequent

Behavioral state	□ Available to interact	□ Unavailable to interact	□ Asleep

#### Clinically Informed Four-Section Structure

The FEEDS is made up of a four-section checklist directed at targeting non-nutritive and nutritive oral-motor skills. The sections are (A) morphological-functional domain; (B) reflexive oral-motor skills; (C) signs of stress or disorganization; and (D) other clinical features. Moreover, an open space for the clinician’s comments is available in order to take notes about parent–infant interaction and other contextual factors which contribute to the infant’s successful or unsuccessful feeding (e.g., the caregiver position with respect to the infant, the infant’s posture). Each session is made up by different sets of items (see Table 2).

**Table 2:** Full list of the Functional Evaluation of Eating Difficulties Scale (FEEDS) items.

Section 1	Item #	Item description	Score
Lips	1	□ available lip seal □ unavailable lip seal	0 2

Tongue	2	□ Protrusion present □ Protrusion absent	0 2
	3	□ Right lateralization present □ Right lateralization absent	0 2
	4	□ Left lateralization present □ Left lateralization absent	0 2
	5	□ Rise response present □ Rise response absent	0 2
	6 7	□ Presence of tremors □ Non-goal directed movements	1 2

Jaw	8	□ Opening present □ Opening absent	0 2
	9	□ Closing present □ Opening absent	0 2

Perioral sensitivity	10	□ Present □ Heightened □ Absent	0 1 2

Intraoral sensitivity	11	□ Present □ Heightened □ Absent	0 1 2

**Section 2**			

Non-nutritive sucking	12	□ Present and mature □ Present and immature □ Present and disorganized □ Dysfunctional □ Absent	0 1 2 2 3

Nutritive sucking	13	□ Present and mature □ Present and immature □ Lack of swallowing/breathing coordination □ present and disorganized □ Dysfunctional □ Absent	0 1 2 2 2 3

Swallowing reflex	14	□ Present □ Ipovalid □ Difficult to trigger □ Absent	0 2 2 4

Saliva control	15	□ Adequate □ Occasional □ Absent □ Presence of stagnation	0 2 4 4

Vomit reflex	16	□ Present and immediate □ Present and delayed □ Heightened □ Absent	0 1 1 2

Cough reflex	17	□ Valid □ Reduced or ineffective □ Absent	0 2 4

**Section 3**			

Autonomous nervous system	18 19 20 21 22 23 24 25 26	□ Skin color changes □ Vital signs variations □ Presence of laryngeal stridor □ Supra- or sub-sternal retractions □ Yawns □ Visceral instability □ Tremors □ Startles □ Clonic movements	4 4 4 4 2 2 2 2 2

**Section 4**			

Gastrointestinal signals	27 28 29 30 31 32 33 34 35 36	□ Irritability □ Rumination □ Arching/Hypertension □ Anomalous body movements □ Bolus stagnation far from meals □ Increased saliva production □ Spit-up □ Refusal of food/stimulations □ Crying during meals □ Abdominal colic	1 1 1 1 1 1 1 1 1 1

Ineffective swallowing	37 38 39 40	□ Stagnation □ Wet voice □ Nasal spit-up □ Frequent detersive acts	4 4 4 4

Penetration/inhalation	41 42 39 40 41 42	□ Cardio-respiratory parameters changes □ Fatigue □ Inspiratory stridor □ Pre-swallowing cough □ Swallowing cough □ Post-swallowing cough	4 3 4 4 4 4

Respiratory difficulties	43 44 45 46	□ Noisy breathing □ Dyspnea □ Laryngeal stridor □ Retractions of jugulum	4 4 4 4

Other signs	47 48 49 50 51	□ Falling asleep □ Discontinued epileptic seizures □ Epileptic seizures □ Bronchiolitis □ Catarrhal obstruction	2 2 2 2 2

##### Section 1—Morphological-Functional Domain

The morphology of the oral district is assessed for its functionality at rest or after adequate stimulation. The following aspects are evaluated: the lip seal at rest or after gentle rhythmic touches on the labial rhyme; the activation of the perilabial muscles after protrusion and stretching stimulations; the potential presence of tremors. Subsequently, the tongue movements are elicited in response to the gentle brush of the inferior lip and gum. The presence of lateral movements, the response to evoked vertical rise of the tongue and the presence of non-goal-directed tongue movements are registered. The mandibular functionality is evaluated observing the opening and closing movements of the mouth. Information on the perioral and intraoral sensitivity are obtained throughout these observations.

##### Section 2—Reflexive Oral-Motor Skills

This section includes the assessment of the presence and quality of non-nutritive sucking, elicited by rhythmic brushing of the assessor index finger on the tongue or the palate. Provided that the infant is safe, nutritive sucking is also assessed in response to liquids or baby food and the presence of the pharyngeal reflex is observed. Other assessments include the control of secretions and the presence of the protective cough and vomit reflexes, which are, respectively, elicited by means of circular external movements on the first tracheal rings and stimulations of the pillars of the soft palate with a tongue depressor.

##### Section 3—Signs of Stress or Disorganization

Every sign of stress and behavioral disorganization of the newborn or infant is registered throughout the procedure. These manifestations might be related to the abnormal activation of the autonomic nervous system and include: changes of skin color, variations of signals parameters (e.g., heart rate, respiratory rate, and oxygen saturation), stridor, yawning, startles, and tremors.

##### Section 4—Other Clinical Features

Moreover, the display of respiratory clinical complications and other indirect signals of dangerous penetration-inhalation which might document swallowing inefficiency are collected throughout the procedure. Lastly, the assessor is expected to note the presence of other clinical events or signals of gastrointestinal diseases which might negatively affect the development of infants’ pre-feeding and feeding skills.

#### Administration

##### Prerequisites

In order for the evaluation to be reliable and valid, the FEEDS administration should occur when the newborn or the infant is in a quiet or active alert state (11). Signals of an incoming drowsy state as well as signs of distress should be absent. Moreover, the infant should be moderately hungry in order to assess the broad spectrum of oral-motor skills. In some cases, more than one session might be necessary in order to describe the best infant performance and to better depict the whole set of the FEEDS items.

##### Procedures

The FEEDS administration is solely permitted to specialized clinical staff (i.e., speech therapist and phoniatrists) and lasts about 30–40 min. The actual duration of FEEDS administration includes a preliminary warm-up period during which the professionals are expected (1) to interact with the parents in order to collect relevant information on the actual context of feeding, developmental notes on the infant developmental trajectories, and the clinical history of the baby; and (2) to interact with the newborn/infant in order to be sure that she/he is at ease and behaviorally ready for the observation.

##### Additional Evaluations

The presence of documented alterations of the oral district morphology and the observed inefficiency and difficulties in non-nutritive and nutritive sucking and swallowing are necessary criteria to signal the need of a rehabilitation intervention. Nonetheless, the professional should note whether the fiberscope exam reports no gastric stagnation and the presence of pre- and post-swallowing bolus loss.

#### Item Coding and Section Scoring

The score attributed to each item is given by defect, with higher scores indicating the presence of alterations, difficulties or problems in each specific feature, structure, or function. The theoretical range of scores has been attributed according to expert clinicians’ judgment of the relative critical importance of the different items to concur for it to negatively affect the development of adequate feeding capacities and skills. The specific scores attributed to the items at the theoretical level include different ranges (e.g., 0–2; 0–4) for different domains. The different weights attributed to the items have been decided on the basis of clinically oriented judgment. For example, difficulties detected by items related to respiration, swallowing, and mechanical coordination are much more critical for the health and development of infants with neurodevelopmental disabilities. As such, these parameters receive higher scores when an impairment or difficulty is detected. A complete list of item scoring is provided in Table 2.

##### Section 1

A score of 0 is given if the specific item functionality is preserved. A score of 1 is given if labial or tongue tremors are observed or if a low threshold for perioral (e.g., the infant starts to fidget when the stimulations occur) or intraoral (e.g., the vomit reflex is evoked even in response to anterior stimulations) sensitivity is observed. A score of 2 is given when the infant does not maintain the lip seal even in absence of external stimulations, there is no response to tongue stimulations, non-goal-directed movements are observed, and opening and closing mouth movements are absent. The theoretical range of this section is 0–20.

##### Section 2

A score of 0 is assigned if the infant presents age-appropriate and adequate non-nutritive and nutritive sucking (i.e., sequences of sucking acts ≥10): the swallowing reflex is present and concurrent with adequate saliva control, the vomit reflex is present and the elicitation of palate arcs is immediate, and the cough reflex follows the stimulations of the first tracheal rings. A score of 1 is given when non-nutritive and/or nutritive sucking is not as mature as for score-0 (i.e., sequences of sucking acts <10): the vomit reflex is delayed, present only in response to posterior pharyngeal stimulations or heightened and elicited by intraoral anterior stimulations. A score of 2 is assigned when the non-nutritive and/or nutritive sucking do not present the typical physiological rhythmicity which characterize adaptive functionality: there might be anomalous movements of the tongue and the jaw, the nutritive sucking is not adequately coordinated with swallowing and breathing in the well-acknowledged 3:1:1 proportion, the swallowing reflex is hardly elicited with partial control of secretions and with the necessity of mechanical suction. A score of 3 is assigned when non-nutritive and nutritive sucking are absent, whereas a score of 4 is given if the basic swallowing reflex is not present, there is a complete lack of secretion control and the need of mechanical suction is constant, with abundant gastric stagnation and no cough reflex. The theoretical range for this section is 0–24.

##### Section 3

A score of 0 is given when no stress signs are detected. A score of 2 is assigned in presence of repeated yawning or labored breathing which might be indirect signs of “air hunger,” visceral instability, general tremors, startles, and clonic movements. A score of 4 is assigned when specific central nervous systems manifestations occur, including changes in skin color, variations of vital signs, and presence of screeching or supra- and sub-sternal retraction. The theoretical range for section 3 is 0–26.

##### Section 4

A score of 1 is assigned in the presence of signs of gastrointestinal diseases (e.g., irritability, rumination, arching, abnormal body movements, bolus stagnation in the oral cavity even far from meal-time, increase of saliva production, spit-up and vomit, refusal of food, persisting crying during meals, abdominal colic) and other clinical signs, such as catarrhal obstructions or bronchiolitis. A score of 2 is given when epileptic seizures are present. A score of 3 is attributed if signs of relevant fatigue emerge during the assessment. A score of 4 is assigned when there are respiratory problems (e.g., noisy breathing, dyspnea, laryngeal stridor, retractions of jugulum), indirect signs of swallowing inefficiency (e.g., stagnation, persistent fuss and crying, nasal spit-up), and potential risk factors for penetration/inhalation (e.g., changes in cardio-respiratory parameters, breathing stridor, pre- and/or post-swallowing cough). The theoretical range for this section is 0–66.

#### Global Scoring

The FEEDS assessment includes a final global score which is computed as the sum of the scores attributed to each item and which ranges from 0 to 136. Despite the fact that the FEEDS was initially developed in order to obtain a quantitative evaluation of newborns and infants capacity to feed, a qualitative evaluation has been also developed in order to provide clinicians with a cutoff which might facilitate important decisions for the health and well-being of at-risk patients. This cutoff is meant to be a proxy to target infants who present the maximum set of risk factors which still guarantee that the start of the rehabilitation pathway for complete weaning and autonomous feeding might be successful.

### Phase 2: Psychometric Characteristics of the FEEDS

#### Sample

**Figure 1:**
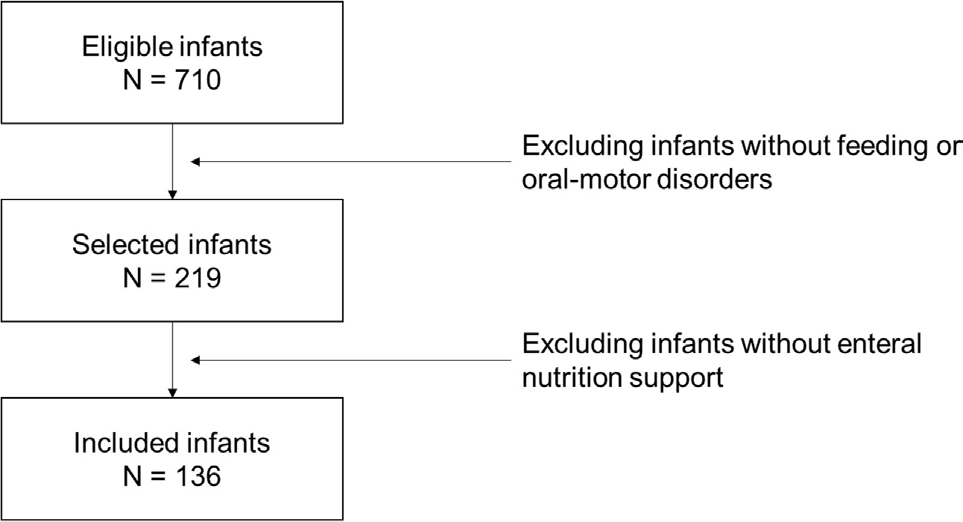
Sample flow chart.

In order to provide adequate information about FEEDS psychometrics, consistency, and reliability, the FEEDS has consecutively been administered to infants hospitalized at the Scientific Institute IRCCS Eugenio Medea from 2004 to 2016. Inclusion criteria were as follows: age between 0 and 12 months at FEEDS assessment and the presence of feeding and/or oral-motor disorders. Figure 1 reports the sampling flowchart. The clinical characteristics of the sample are resumed in Table 3.

**Table 3:** Characteristics of the present sample.

Scalar measures	Mean	SD	Min	Max
Gestational age at birth (weeks)	37.31	4.10	24.70	41.60
Birth weight (grams)	3,072.42	914.24	495.00	4,480.00
Apgar at minute 1	6.59	3.31	0.00	10.00
Age at the FEEDS assessment (months)	5.45	3.49	0.00	12.00

**Categorical measures**	***N***	**%**		

Gender				
Males	72	52.9		
Females	64	47.1		
Small for gestational age				
Yes	6	4.4		
No	130	95.6		
Preterm birth (<37 weeks)				
Yes	45	33.1		
No	91	66.9		

**Diagnoses**	***N***	**%**		

Brain infections	3	2.2		
Chromosomopathy	22	16.2		
Extreme prematurity	15	11.0		
Genetic syndrome (involving the CNS)	10	7.4		
Genetic syndrome (non-involving the CSN)	22	16.2		
Isolated anatomic malformations	3	2.2		
Malformation syndrome	8	5.9		
Metabolic syndrome	5	3.7		
Myopathy	2	1.5		
Neonatal asphyxia	38	27.9		
Diagnosis not available	8	5.9		

#### Plan of Psychometric Diagnostic Analyses

The reliability of the FEEDS has been assessed according to (a) internal consistency, measured by means of the standardized Cronbach’s alpha and (b) inter-rater agreement, measured by means of inter-class correlation coefficient (provided as % of agreement) on a subset of FEEDS assessment which were done by two different coders who were unaware of reciprocal scores. Moreover, in order to provide a clinically relevant and statistically sound cutoff, the receiver operating characteristics (ROC) curve analysis has been used. The analysis has been done using SPSS IBM Statistics 21.

#### Reliability

The standardized Cronbach’s alpha was 0.91, which represents an index of optimal internal consistency of the instrument. The inter-class coefficient was 0.99, documenting a relevantly high reliability among different assessors.

#### Cut-Off Estimation

**Figure 2:**
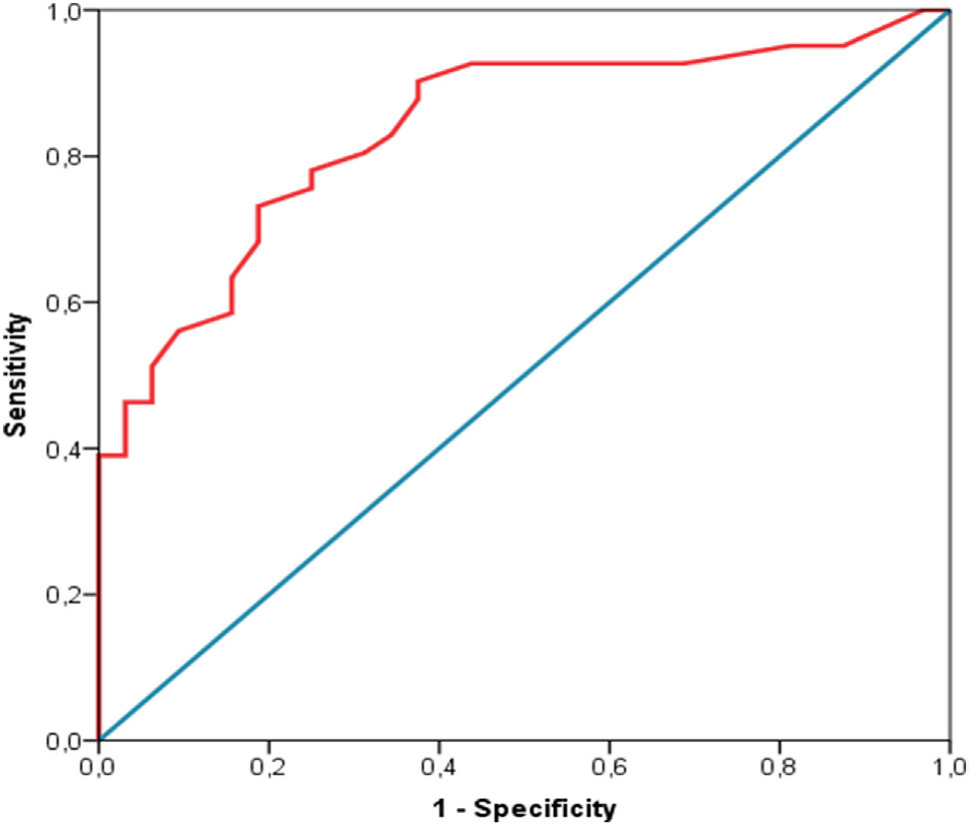
ROC curve analysis for the FEEDs cut-off estimation.

A conservative approach has been applied, and both specificity (0.81) and sensitivity (0.76) have been balanced setting the cutoff at 16.50 (Figure 2). A different cutoff, which maximizes sensitivity might be set at 13.50, despite the risk of false-positive raise at 0.38.

## Advantages and Limitations

### Advantages

The FEEDS presents several advantages compared to other available tools in clinical practice. First, it has a documented protocol of methodological validation carried out in a relatively large sample of infants who represent a comprehensive and representative target of the population of infants with neurodevelopmental disabilities involving the biomechanical impairment of swallowing and feeding. Second, the FEEDS protocol is made up a multidimensional checklist which is easily administrable and the protocol is detailed enough to make the procedure replicable by other clinicians or groups. Third, this tool has been specifically developed by clinicians and researchers who have well-acknowledged expertise with infants who present neurodevelopmental disorders and indeed it is well-grounded in clinical practice and responds to specific and highly relevant clinical needs. Fourth, the final score is indexed according to a statistically defined cutoff which is helpful to help clinicians in decision making for what concerns the support of weaning and the initiation of the most appropriate oral-motor rehabilitation journey for each infant. In other words, this tool supports a tailored and individualized rehabilitation of biomechanical impairments of swallowing and feeding in infants with neurodevelopmental disorders.

### Limitations

Despite the FEEDS has specific and relevant clinical advantages, it should be noted that sometimes the clinical complexity of these infants may affect the opportunity to administer this protocol. Nonetheless, this limitation applies to all the oral-motor evaluation of infants with severe and complex neurodevelopmental disorders. Nonetheless, this limitation is partially counterbalanced by the possibility that the clinician may integrate the score obtained by an infant during the FEEDS examination with clinical insights. For example, in a very young and severely impaired infant, the FEEDS score might indicate that he/she can be weaned without the use of specific rehabilitation support, but the clinicians also know that he has frequent seizures every 2–3 min. In this case, the score obtained from the FEEDS is not enough and case-by-case clinical judgment is required to support the final decision making for the healthcare journey of that infant. Moreover, and even for the abovementioned reason, the FEEDS should be better administered by a specialized speech therapist who works within a multidisciplinary clinical team.

## Ethics and Dissemination

### Ethics

This study was carried out in accordance with the recommendations of Scientific Institute IRCCS Eugenio Medea with written informed consent from all subjects. All subjects gave written informed consent in accordance with the Declaration of Helsinki. The protocol was approved by the Scientific Institute IRCCS Eugenio Medea.

### Dissemination

The present study protocol presents the development and the preliminary assessment of psychometric properties of the FEEDS. The analyses document adequate reliability and internal consistency of the instrument. Nonetheless, greater samples will be obtained for further methodological and psychometric evaluations, including a dimension-reduction statistics (i.e., principal component analysis) to evaluate the clinically grounded subdivision of the FEEDS into four different sections. Moreover, the ability of the FEEDS assessment to be reliably predictive of developmental outcomes of at-risk infants and to precociously target infants who are potentially ready to be trained for autonomous feeding will be the focus of a longitudinal clinical trial which is actually ongoing. The findings of further clinical assessments and scientific investigations with the FEEDS will be object of scientific publications on indexed and impacted scientific journals in the field of pediatrics, nursing science, and rehabilitation and they will be presented at international congresses.

## Ethics Statement

This study was carried out in accordance with the recommendations of Scientific Institute IRCCS Eugenio Medea with written informed consent from all subjects. All subjects gave written informed consent in accordance with the Declaration of Helsinki. The protocol was approved by the Scientific Institute IRCCS Eugenio Medea.

## Author Contributions

RB conceived the study in consultation with DS and AC; DS and AC provided the clinical expertise necessary to the development of the FEEDS items and scoring; LL sampled infants for preliminary FEEDS assessment; DS and LL conducted FEEDS coding, and AC provided supervised judgment in cases of coding disagreement; LP performed statistical analyses. All authors contributed to the drafting of the work and/or revised it critically and all of them approved the final version of the manuscript.

## Conflict of Interest Statement

The authors declare that the research was conducted in the absence of any commercial or financial relationships that could be construed as a potential conflict of interest.

## Footnotes

^1^Longoni L, Cavallini A, Sacchi D, Provenzi L, Borgatti R. Predictors and outcomes of the neonatal oral motor assessment scale (NOMAS) performance: a systematic review. Eur J Pediatr (Forthcoming).
